# Leveraging Multi-Task Learning to Cope With Poor and Missing Labels of Mammograms

**DOI:** 10.3389/fradi.2021.796078

**Published:** 2022-01-11

**Authors:** Mickael Tardy, Diana Mateus

**Affiliations:** ^1^Ecole Centrale de Nantes, LS2N, UMR CNRS 6004, Nantes, France; ^2^Hera-MI SAS, Saint-Herblain, France

**Keywords:** breast cancer, mammography, classification, multi-task learning, missing labels, uncertainty

## Abstract

In breast cancer screening, binary classification of mammograms is a common task aiming to determine whether a case is malignant or benign. A Computer-Aided Diagnosis (CADx) system based on a trainable classifier requires clean data and labels coming from a confirmed diagnosis. Unfortunately, such labels are not easy to obtain in clinical practice, since the histopathological reports of biopsy may not be available alongside mammograms, while normal cases may not have an explicit follow-up confirmation. Such ambiguities result either in reducing the number of samples eligible for training or in a label uncertainty that may decrease the performances. In this work, we maximize the number of samples for training relying on multi-task learning. We design a deep-neural-network-based classifier yielding multiple outputs in one forward pass. The predicted classes include binary malignancy, cancer probability estimation, breast density, and image laterality. Since few samples have all classes available and confirmed, we propose to introduce the uncertainty related to the classes as a per-sample weight during training. Such weighting prevents updating the network's parameters when training on uncertain or missing labels. We evaluate our approach on the public INBreast and private datasets, showing statistically significant improvements compared to baseline and independent state-of-the-art approaches. Moreover, we use mammograms from Susan G. Komen Tissue Bank for fine-tuning, further demonstrating the ability to improve the performances in our multi-task learning setup from raw clinical data. We achieved the binary classification performance of *AUC* = 80.46 on our private dataset and *AUC* = 85.23 on the INBreast dataset.

## 1. Introduction

Breast cancer is one most prevalent types of cancer worldwide (1)^1^ and, therefore, an important healthcare concern. Significant efforts are dedicated to breast cancer screening (2), as early detection allows to increase the chances of recovery (3). Screening usually begins with a clinical exam followed by an imaging examination, with mammography as the most common first choice (4). Generally, the mammography interpretation further guides patient care, which can be a regular follow-up, or additional examinations if mammography reveals any signs of pathology. Other imaging examinations, such as Ultrasound (US) or Magnetic Resonance Imaging (MRI) can also be performed during the clinical investigation. However, they are often secondary to mammography, i.e., they are used for diagnostic purposes when the clinical examination and mammography are not sufficient. In this work, we focus on early breast cancer screening and diagnosis. Therefore, our method targets mainly mammography. Nevertheless, it can be applied to other types of imaging as well.

Regular mammography screening starts between the age of 40 and 50, depending on the regional guidelines (4, 5), and is performed every 2 years. With a growing number of people having access to public and private healthcare, screening generates a substantial workload for healthcare practitioners, in particular for radiologists. Moreover, new imaging modalities, such as Digital Breast Tomosynthesis (DBT) (6) or Automated Breast Ultrasound (ABUS) (7) improve precision and allow for earlier cancer detection at the cost of longer interpretation time. Hence, the clinical community has shown an interest in tools able to facilitate routine diagnosis. In response, Computer-Aided Detection (CADe) and Diagnosis (CADx) methods have gained popularity, in particular with the recent emergence of deep-learning-based methods (8–11). Such CAD tools are intended to assist the radiologist during image interpretation by providing detection guidance and evaluating the likelihood of cancer, aiming to reduce interpretation time. Although a general adoption is yet to come (8, 12), there is a trend toward a larger acceptance of CAD software as a helper tool in clinical practice (13, 14). Today, common breast cancer CADx solutions provide a cancer-probability- or risk-based score of malignancy. Following the radiology guidelines, such as American College of Radiology (ACR) guidance (15), that suggests listing and classifying all identifiable findings according to the probability of cancer (from 0 to >95%).

Most recent CAD algorithms (9, 16–18), based on supervised Deep Learning (DL) approaches, rely on a set of samples for training. The model's success during test time will largely depend on this dataset, which should be **representative** of the variations, clinically **relevant**, and preferably include **explicit** ground truth annotations. As we discuss next, these requirements are challenging in the context of mammography imaging analysis.

First, access to clinical data is restricted due to patient privacy concerns. Healthcare providers holding imaging databases are not allowed to share the data, either for free or for a fee, without patients' consent. That is, patients shall be informed and confirm that they agree to their data being used for research or software development purposes. Hence, a protocol compliant with regulations needs to be carefully designed before sharing images with third parties.

Second, clinical data from screening protocols are strongly imbalanced. With breast cancer prevalence in a range between 1 and 2%^1^, screening imaging databases are mainly composed of negative cases while the true positive (biopsy-proven) cases are generally under-represented.

Third, ground truth for the images is not always easy to collect. Generally, each clinical case is composed of a set of images and a descriptive clinical report. Often, reports contain information about the images (number and type of acquisitions), physiological details (breast density), and the most important information about the diagnosis. Depending on radiologists' practice in each site, the level of details may significantly vary. Also, while the clinical report of a mammography usually contains an ACR classification indicating the probability of cancer, the actual confirmation of the malignancy may not be available; for instance, the biopsy reports can be stored in a different database (e.g., paper hard-copies), or be effectively missing. Besides, there is a certain number of false-negative mammography exams, estimated to 10 − 15% in (9, 19). Hence, despite the substantial volume of screening data, image databases relying on labels from clinical repots can be poorly and sometimes erroneously annotated, leading to significant label uncertaint ies.

The issues above make the design and training of deep learning CAD solutions difficult. To better exploit raw clinical databases, in this work, we propose a Multi-Task Learning (MTL) approach allowing us to maximize the quantity of data used for training, without the need to carefully curate sample annotations. To this end, we propose to simultaneously train a neural network for multiple tasks relevant to mammography. Our main purpose is to mitigate the data (epistemic) uncertainty by encouraging the network to learn common features despite missing or noisy labels, thereby improving the performances of each particular task. Moreover, our training strategy allows for a better shaped latent space, contributing to the explainability of the prediction. Finally, our design has the potential of capturing uncertainty as in (20) on several tasks simultaneously, further increasing the safety of the solution.

Our multitask model focuses on five tasks. First, we set the binary malignancy prediction as a primary task. Next, we define four auxiliary tasks: (1) 6-class ACR prediction, (2) 4-class breast density prediction, (3) 2-class view angle prediction, and (4) image reconstruction. To the best of our knowledge, we are the first to propose the combination of these five tasks in a single model.

### 1.1. Related Work

Deep learning applied to mammography imaging has been broadly studied in past years (21–23). Recent works include deep neural networks for classification (17, 18, 20, 24), detection (16, 25), and segmentation (26–28).

Several successful methods for breast cancer classification from mammograms have been recently proposed. Shen et al. (17) describe a binary-classification method trained on two datasets, CBIS-DDSM (29) and INBreast (30) reaching *AUC* = 95.00 on selected validation samples from INBreast. Ribli et al. (16) approach the classification task with a detection method. The detector is trained on two datasets: DDSM (31) and privately collected by the authors. Both datasets includes manually delineated Regions of Interest (ROI) around lesions. Using the entire INBreast dataset for validation (16) claim a similar *AUC* = 95.00. Other works, such as (18, 32, 33) use substantially larger private datasets for training and testing, with AUC scores of 89.50, 93.00, and 94.50, respectively. Both (18) and (32) rely only on image-wise labels (i.e., without ROI). Wu et al. (18) classify mammography cases (composed of four views) by combining features extracted from each view. Shen et al. (32) perform image-wise classification and introduce an additional step that evaluates patches extracted from the image using saliency map to get the most relevant patches. Alternatively, Lotter et al. (33) train a network using explicit pixel-wise ground truth for detection, before fine-tuning with image-wise labels on a larger dataset.

There is a trend toward algorithms processing multiple mammography views of the same patient simultaneously (34–38). In clinical practice, two views of a breast, called Craniocaudal (CC) and Medio-lateral Oblique (MLO), are usually acquired from two different angles. These views seek to compensate the tissue superimposition when projecting the 3-dimensional breast onto a 2-dimensional mammogram. Building an algorithm relying on multiple views has the potential to improve the performance. However, such algorithms may fail when an abnormality is seen from one view only (39). Moreover, a case-wise algorithm requiring the images of both breasts may fail to operate on cases with mastectomy (i.e., one breast is missing). Hence, instead of processing several images simultaneously, we we use the view information to train a network to distinguish the two views as an auxiliary 2-class objective.

Breast density is a risk indicator for breast cancer (40). Therefore, developing methods for density classification has also attracted the interest of the community (41–43). Recently, Arefan et al. (44) proposed a neural-network-based method using density to predict the risk of cancer development. However, the authors relied on 224x224 images, which is insufficient to detect cancer-related clinical features being sometimes smaller than 1*mm* (15). In our case, we propose to learn the density representation as another auxiliary classification task, using the 5th edition Breast Imaging-Reporting And Data System (BI-RADS) density classification grid, and to perform this task on high-resolution images.

Our method is built on the MTL strategy (45) previously studies in the context of other medical imaging applications(46, 47). State-of-the-art methods often implement MTL as a combination of detection and classification tasks (37, 48–50). Other works refer to the MTL as a means of pre-training on unrelated on distantly-related tasks (51, 52). In our case, we propose to train an MTL algorithm from scratch on multiple classification tasks relevant to mammography, namely breast cancer, view angle, breast density, and probability of cancer. To this end, we rely on image-wise labels generally available from clinical practice (i.e., clinical case reports), without the need for explicit pixel- or region-wise ground truth.

From the architecture standpoint, we propose to combine several techniques successfully used on other modalities. Our architecture is similar to a Y-net (53). H owever, our network yields multiple predictions from the bottleneck instead of one unique output as in the case of (53). Similar to (25), we propose the fusion of features at multiple levels, preventing from choosing one particular (e.g., last) feature level. In this way, we leave the network select the features relevant to a given task automatically during learning.

Finally, we propose to introduce a measure of uncertainty in the training process. Contrary to (54), who quantify the uncertainty of output predictions, we propose to use the uncertainty estimates within the optimization function similar to (55). However, we propose to rely on prior knowledge about the dataset to scale the uncertainty, instead of using the uncertainty coming from the network as in (55). In a way, our approach relates to the work of (56), who use inter-rater agreement as an uncertainty indicator.

## 2. Methods and Materials

### 2.1. Method

In this work, we focus on the problem of mammography binary breast cancer classification in the context of data with heterogeneous annotations. First, we have a limited number of well-annotated samples with gold-standard-confirmed ground truth. Second, the available samples also have labels of other classes, such as density (i.e., BI-RADS), cancer probability (i.e., ACR), and view angle. Third, we have access to an extended dataset with images having labels of other classes only, without confirmed breast cancer classification. We aim at maximizing the source s of knowledge using most of the available data for the training of an image classifier.

We address the problem of poorness and uncertainty of the ground truth labels with an MTL approach. Let *I* ∈ ℝ^*H* × *W*^ be an input mammography and let each image *I* have at most *T* labels (one per classification task ) leading to a target vector **y** = [*y*_1_, ..., *y*_*T*_]. For each task, the label *y*_*t*_ is defined as *y*_*t*_ ∈ {0, *C*_1_, *C*_2_, ..., *C*_*t*_} for a *C*_*t*_-class classification, where the label *y*_*t*_ can belong to one of the *C*_*t*_ classes, or be missing (i.e., *y*_*t*_ = 0). Moreover, each label *y*_*t*_ can be associated to an uncertainty score *u*_*t*_. This score allows taking into account the low confidence of a label. There can be several scenarios determining the value of *u*_*t*_, for example, (i) the expertise of the annotator, e.g., labels generated by a junior-level radiologist; (ii) the impreciseness of labels extracted from the clinical report, e.g., “density between B and C”; (iii) the low confidence of a reported diagnosis, e.g., a malignant case may have a negative mammogram. For all the above cases, the uncertainty values are bounded to *u*_*t*_ ∈ [0, 1], and there is one uncertainty estimation per task leading to a vector **u** = [*u*_1_, ..., *u*_*T*_].

We define a trainable classifier *f*(·, θ) predicting the *T* classes for the input image *I*, where θ are trainable parameters. Having *T* classification outputs [ŷ_1_, ..., ŷ_*T*_], we define *T* classification loss functions L_*cls*_{1, ..., *T*}__. To take into account both the missing and uncertain labels, we introduce a loss-weighting function *W*(**y**, **u**) combining the individual task losses into the global loss L_*CL*_ as follows:


(1) $$\begin{array}{l} L_{CL}=\overset{T}{\underset{t=1}{\sum }}w_{t}L_{{cls}_{t}}\left(y_{t},{ŷ}_{t}\right), \end{array}$$

where *w*_*t*_ is defined as:


(2) $$\begin{array}{l} w_{t}=W\left(y_{t},u_{t}\right)=\{\begin{array}{ll} 0 & y_{t}=0\\ 1-u_{t} & \text{otherwise} \end{array} \end{array}$$

Following Equations (1) and (2), for a given image *I* whose label *y*_*t*_ is available, the loss L_*cls*_*t*__ is enabled and weighted with the inverse of the label's uncertainty score without any additional normalization. If the label is unavailable (*y*_*t*_ = 0), the loss L_*cls*_*t*__ is disabled for that sample.

To further maximize the knowledge used by the classifier, we add a reconstruction task as a means of implicit regularization (57). Unlike several state-of-the-art works proposing a segmentation output (53) as an addition to classification, we prefer the reconstruction task since it requires neither explicit ground truth nor custom losses as in (28). Hence, the *f*(·, θ) function yields an auxiliary output image Î ∈ R^*H* × *W*^ on top of the classification predictions [ŷ_1_, ..., ŷ_*T*_]. The global loss function, including the reconstruction loss, is defined as follows:


(3) $$\begin{array}{l} L=L_{rec}\left(I,Î\right)+\overset{T}{\underset{t=1}{\sum }}w_{t}L_{{cls}_{t}}\left(y_{t},{ŷ}_{t}\right) \end{array}$$

**Figure 1:**
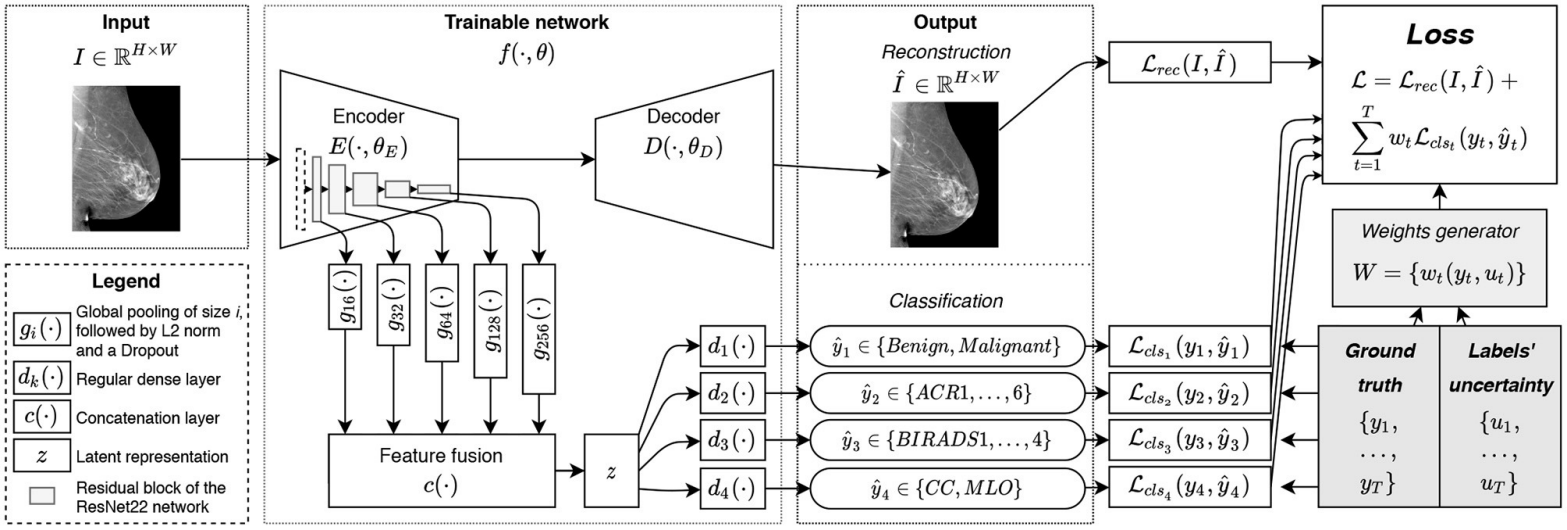
The overview of the proposed method: the image *I* is fed to the network *f*(·); the encoder *E*(·) allows to extract the latent representation *z*(·) used for classification tasks {ŷ_*t*_}; the decoder *D*(·) is trained to reconstruct the image *I* from its latent representation *z*(·) leading to the auxiliary output Î.

The overview of the proposed method is illustrated in Figure 1. Training the neural network with the loss from Equation (3) allows updating most of the parameters of the network from every sample while requiring very few data filtering beforehand. Indeed, the reconstruction task is systematically feasible regardless of the available ground truth. Amongst the classification tasks, the view angle is almost always available, with rare mislabeling often due to acquisition mishandling. Fewer density labels are available, but they can be crowdsourced (58) from junior radiologists. Finally, ACR and cancer annotations are more challenging to collect and often unavailable. However, as we demonstrate experimentally in section 3, our method relies on the auxiliary tasks with easier to collect labels to improve the classification performance for the more challenging ACR and benign/malignant classification tasks.

### 2.2. Architectural Design

For the implementation of our trainable function *f*(·, θ), we rely on a hourglass auto-encoder architecture implemented as a Deep Neural Network (DNN), as in (28). In practice, *f*(·, θ) is composed of an encoder *E*(·, θ_*E*_), a decoder *D*(·, θ_*D*_). To facilitate our MTL formulation we include in *f*(·, θ) *T* classification functions *d*_*t*_(·) that convert the latent representation *z* of an image into the predictions ŷ_*t*_. Finally, we rely on a multi-level feature extraction in *E*(·, θ_*E*_), as we explain next.

The encoder *E*(·, θ_*E*_) is implemented as ResNet22 (18, 28, 32). The implemented neural network has 5 levels of residual blocks, each with the following numbers of filters: 16, 32, 64, 128, and 256 in the convolutional layers. We use two residual blocks at each level with two convolutional layers each. As in (28), we used depth-wise separable convolutions (59) instead of regular ones, significantly reducing the number of parameters to train. We replaced the ReLU activations with LeakyReLU and used instance normalization as it better fits the reconstruction task of the decoder (60).

With an MTL approach comes the question of what features are relevant to each task (45). For instance, the view angle classification is relatively simple and may not require deep features: detecting the pectoral muscle on an image is enough. Density classification is harder, while malignancy classification is the most complexcalling for deeper features. To avoid making a restrictive choice of the features to be used for a given classification task, we perform feature fusion from the five levels of the encoder *E*(·). For the consistency of the extracted features, at the end of each level of the ResNet22 encoder we add a **feature generator**, denote d as *g*_*i*_(·) (see Figure 1), composed of a Global Average Pooling (GAP) layer, L2-normalization, and a dropout layer, hence, yielding the features in a normalized scale. The features from *g*_*i*_(·) are then fused with a concatenation function *c*(·) and result in latent representation *z*. Considering the number of the filters at each level of the network, the total size of feature vector *z* is ℝ^496^.

The classification functions *d*_*t*_(·) are implemented as regular dense layers converting the latent representation *z* to a given prediction ŷ_*t*_. In our implementation, we used only one dense layer to reduce the feature space of ℝ^496^ to the dimensionality of the given task (see section 2.3). This choice allows to decrease the complexity of the network and to prevent eventual overfitting.

The decoder *D*(·, θ_*D*_), used only for the reconstruction task, is implemented as a narrower version of an upside-down ResNet22. T o reduce the number of trainable parameters, only one residual block is used at each level of the decoder (i.e., two stacked convolutional layers and a skip connection). To maximize the information flowing through the bottleneck of our hourglass architecture, unlike (28), we did not use any skip connections between the encoder and decoder. We rec kon that such a choice penalizes the quality of the reconstruction. However, we are mainly interested in a meaningful representation *z*, while reconstruction remains an auxiliary task useful for efficient weights initialization (61) and implicit regularization (57).

### 2.3. Classification Tasks

In this work, we restrict the scope to four meaningful-to-mammography classification tasks:

- binary breast cancer classification (*breast*_bin_),
- 6-class ACR cancer probability prediction (*ACR*) (15),
- 4-class BI-RADS density classification (*BIRADS*) (15), and
- 2-class view angle prediction (*view*).

The *breast*_bin_ classification relies on the confirmation of the malignancy either with a histopathological examination or with a follow-up exam. Only samples having positive biopsy are classed as “malignant”. Otherwise, if the re is a negative biopsy, or a negative follow-up, the case is considered “benign.”

The *ACR* classification includes six classes according to the ACR grid as follows:

1. no identifiable finding,
2. all findings are benign,
3. below 2% of the probability of malignancy,
4. between 2% and 94% of the probability of malignancy,
5. more than 95% of the probability of malignancy,
6. confirmed malignant cases.

We do not use the label “0” standing for the lack of imaging to provide the diagnosis.

**Figure 2:**
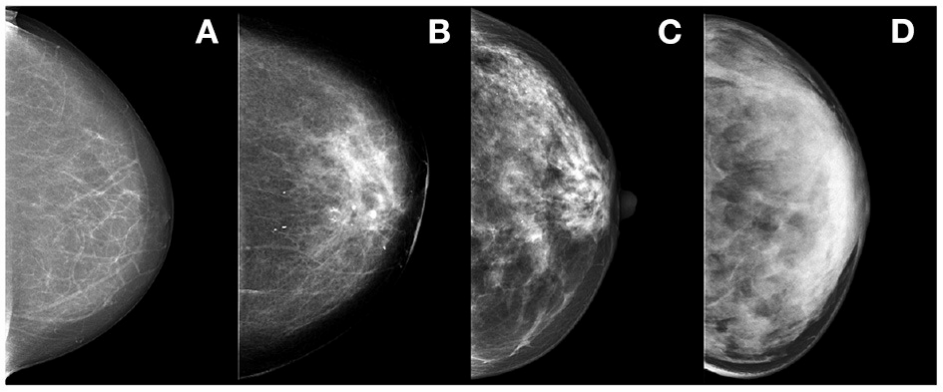
Illustration of the BI-RADS density classes, from left to right: classes **(A–D)**.

The *BIRADS* classification relies on the 5th edition BI-RADS 4-class density assessment guidelines standing as follows:

A fatty,
B scattered fibro-glandular,
C heterogeneously dense; and
D extremely dense (see Figure 2).

**Figure 3:**
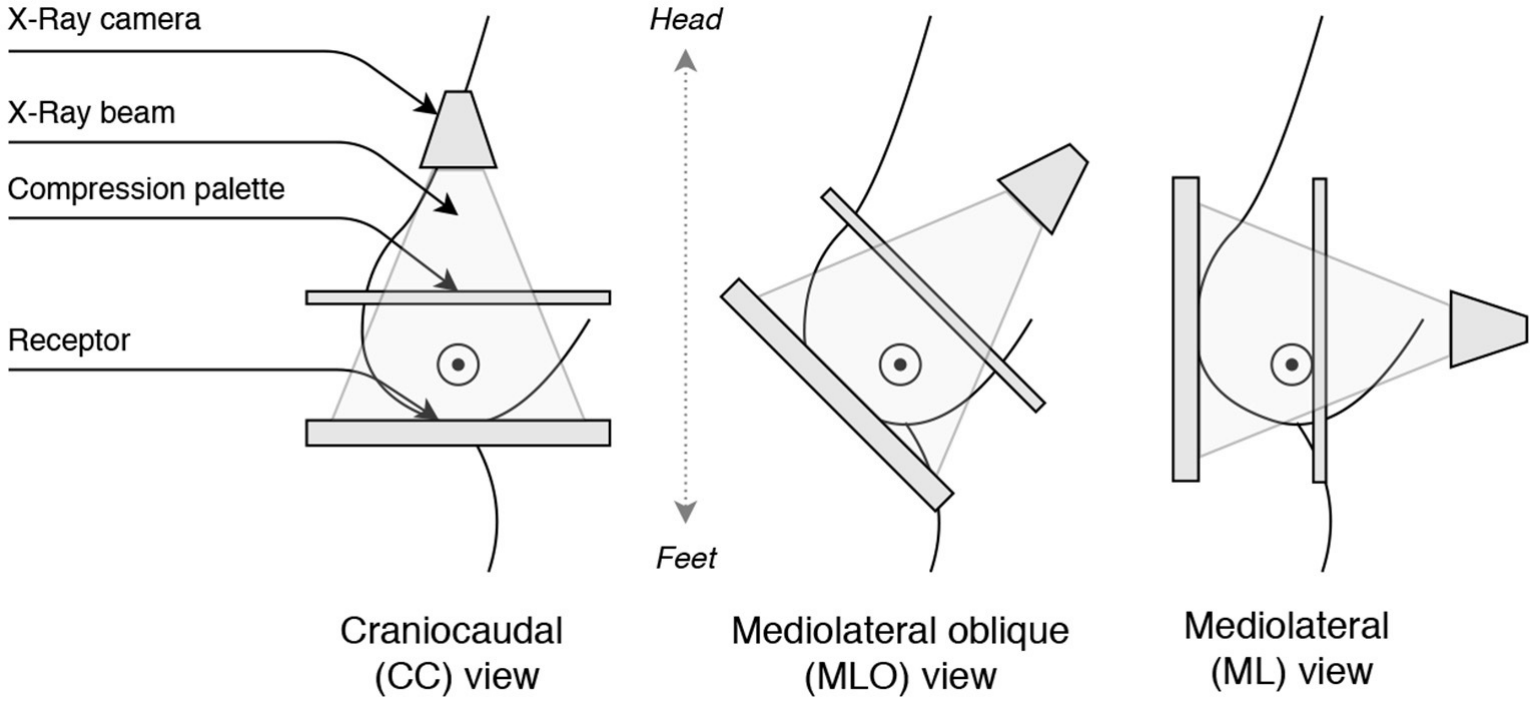
Illustration of the breast view angles: Craniocaudal (CC), Mediolateral oblique (MLO), and Mediolateral (ML).

Finally, the view classification relies on the view angle coming from the X-ray camera position. Most commonly, two view angles are acquired (see Figure 3):

- CC
- MLO

The Medio-lateral (ML) views are rarely acquired, so we restrict the problem to a binary classification to prevent class imbalance. Considering acquisition similarities of ML and CC views (i.e., both views depict the pectoral muscle less than MLO), we combine ML and CC samples together.

For the classification loss functions L_*cls*_*t*__ we use Cross-Entropy losses. For the reconstruction L_*rec*_ we use Mean Squared Error (MSE) loss function. We did not use any loss weighing other than *w*_*k*_(*y*_*k*_, *u*_*k*_).

### 2.4. Implementation Details

The code was implemented using Keras (62) and Tensorflow (63). For the training, we used Adam optimizer with the learning rate of 1*e* − 3, taking into account the sum of five losses used to train the network. The numbers of epochs in different experiments (see section 3.3) vary and mainly depend on whether the training is performed from scratch or fine-tuned. We set the dropout rate to 0.5. In all experiments, except for fine-tuning (see section 3.3.5), the neural network is trained from scratch and the weights initialized with Xavier method (64). No additional hyperparameter was used to balance the different loss terms, i.e., balancing factors are fixed to 1.

## 3. Experimental Validation

### 3.1. Datasets

The experimental validation relies on three datasets coming from different populations, locations (countries), and mammography systems' vendors.

First, we use a private multi-vendor dataset composed of 2,520 Full Field Digital Mammography (FFDM) images from four different vendors, namely Fujifilm, GE, Hologic, and Planmed. It contains 1,271 benign and 1,249 malignant mammograms. All the images were annotated with the labels for the four considered classification tasks: binary cancer classification, cancer probability, density, and view angle classes. We excluded cases with implants and clips, as well as post-surgical malignant cases. In the following, we refer to this dataset as **HMI** and the images of this dataset as *I* ∈ *D*_HMI_.

Second, we use the publicly available INBreast dataset (30) composed of 410 FFDM images from a Siemens mammography system. Similar to the **HMI** dataset, the images were annotated with the labels for the four considered classification tasks. We count 100 malignant images and 310 benign images (including ACR3 class). This dataset is denoted as **INB** and its images as *I* ∈ *D*_INB_.

Finally, we use data from the Susan G. Komen Tissue Bank at the IU Simon Cancer Center (65). This dataset is composed of ≈ 16*K* donated mammograms coming mainly from healthy (i.e., negative biopsy patients). The images do not systematically have labels of all four tasks, often missing cancer classification and sometimes missing the probability of cancer or the density class. However, most of the cases are label ed with density and/or cancer probability. The images come mainly from Hologic mammography systems, ≈ 10% from Fujifilm and GE systems, and the remaining from other lower represented systems (under 5%). We did not apply any filtering of the samples. We refer to this dataset as **VTB** and the images of this dataset as *I* ∈ *D*_VTB_.

The distribution of samples over classes and tasks is given is given in Table 1.

**Table 1:** Composition of datasets *D*_HMI_train__, *D*_HMI_test__, *D*_INB_, and *D*_VTB_, per task and per class as well as the total amount of samples.

	**breast_**bin**_**		**ACR**						**BIRADS**				**view**		**Total**	
	**0**	**1**	**1**	**2**	**3**	**4**	**5**	**6**	**A**	**B**	**C**	**D**	**CC**	**MLO**	**Images**	**Patients**
*D*_HMI_train__	1,016	1,000	397	619	11	186	284	508	372	925	602	117	1,210	806	2,016	997
*D*_HMI_test__	255	249	96	159	2	45	75	123	91	221	159	33	296	208	504	409
*D*_INB_	310	100	67	220	23	43	49	8	136	147	99	28	204	206	410	115
*D*_VTB_	NA	NA	3,152	1,994	94	102	0	22	667	2,489	2,082	198	2,791	2,669	5,460	606

### 3.2. Image Preprocessing and Augmentation

**Figure 4:**
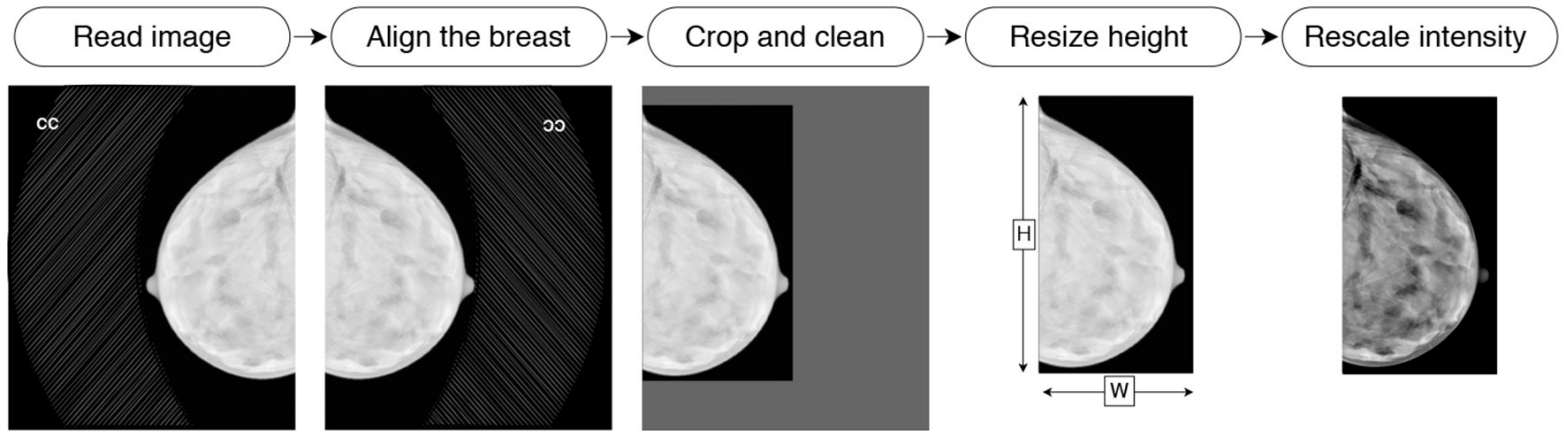
Illustration of the preprocessing pipeline: reading, flipping, cleaning, cropping, resizing, and intensity rescaling.

Before feeding images into the neural network, we preprocess them in the following way. First, the images of the right breasts are horizontally flipped to align the object to the left of the canvas. Then, the background and the embedded labels are removed from the image by (i) determining the intensity value of the background, (ii) identifying the biggest isolated object on the image with binary thresholding, and (iii) setting to zero the pixels of smaller objects (i.e., embedded labels) and the background. Then the image is cropped to the bounding box around the breast. The cropped image is resized to 2, 048 pixels height. The width is padded with black pixels to allow the image to be reconstructed after being passed through the bottleneck of the hourglass architecture. Since our bottleneck is of size $\frac{H}{64}\times \frac{W}{64}\times 256$, we pad the width to ensure the remainder of $\frac{W}{64}$ is zero. The fully convolutional nature of the network (i.e., ResNet) allows to process input images of flexible dimensions. Finally, the intensity values are rescaled to the range of [0, 1]. We illustrate the pre-processing pipeline in Figure 4. All operations are deterministic and integrated into the pipeline in an end-to-end manner.

We apply several augmentation techniques at train time, including (i) random horizontal and vertical translation, (ii) random zoom, and (iii) random vertical flipping. Each image can be modified with none, one, or several augmentations with a probability of 0.5 for each technique. Our main training dataset (i.e., *D*_HM_I__train__) contains limited number of samples (see Table 1). Hence, to prevent the overfitting we observed during our first experiments, we applied data augmentation techniques. We rely on the state-of-the art augmentation techniques described in (16, 17). However, we avoid shearing to prevent artificial deformations, and we did not use horizontal flipping to keep the overall shape consistency as in (18).

### 3.3. Experimental Setup

**Figure 5:**
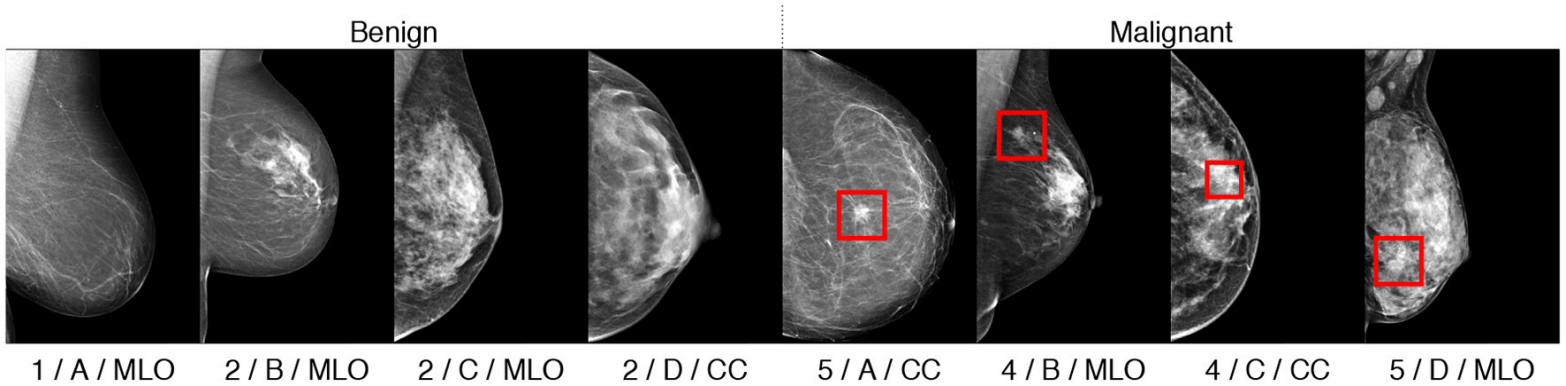
Illustration of classified samples with their annotations in format “ACR/BIRADS/view.” Malignant findings, if any, are indicated with rectangular bounding boxes.

To explore and evaluate our multitask method we perform several experiments and ablation studies that show the interest of each task in improving the joint latent representation and consequently, the overall classification performance. We use the **HMI** dataset for training and testing, split with a 80/20 ratio. We separated the images breast-wise, i.e., images of the same breast belong to the same subset. We note that several the patients had only one view available for a malignant breast. We refer to the train images as *D*_HMI_train__ and to the test images as *D*_HMI_test__. Similar to (16), we use the **INB** dataset for validation only; that is, we do not train the networks on the **INB** dataset. Finally, we use the **VTB** dataset for the fine-tuning of our network. These datasets were fully or partially annotated for the 4 classification tasks (*breast*_bin_, *ACR*, *BIRADS*, *view*), as mentioned in section 2.3. An illustration of samples and their multitask classes is given in Figure 5.

We evaluate the performance of the breast classification and the view angle with the tasks with Area Under the Receiver Operator Curve (AUC). The ACR and BI-RADS classification tasks are evaluated with weighted accuracy metrics to compensate for the class imbalance.

Unless stated otherwise, the training is performed for 100 epochs from scratch. We calculate the average and the standard deviation of the metrics over the 10 top-performing epochs on the *D*_HM_I__test__ dataset.

#### 3.3.1. State-of-the-Art Comparison

To compare our method to the state-of-the-art, we evaluate the performance of several approaches on the *D*_HMI_test__ and *D*_INB_ datasets following the same experimental setup. First, we set as baseline network a ResNet22, denoted as “Baseline,” similar to the encoder *E*(·) but without multi-level feature fusion and trained for a single binary classification task. We train this baseline model from scratch on *D*_HMI_train__. We also compare the ResNet22 implementation from (18), denoted as “Wu et al.” Unlike ours, this implementation uses regular convolutions and contains additional dense layers after the GAP. We used the pre-trained image-wise model, made publicly available by the authors^2^ after training on ≈ 1*M* images. Then, we compare to an ensemble approach, denoted as “Ensembles.” The ensemble combines the top-performing models of the baseline ResNet22 architecture from 5 runs of training from scratch with 5 different random seeds. Finally, we compare to the Monte-Carlo (MC) dropout method (66), denoted as “MC-Drop,” running 10 forward passes of the baseline network with a dropout of 0.5 at test time and computing the average over the predictions.

We report the results on the two test datasets (*D*_HMI_test__ and *D*_INB_). We also report results for the auxiliary tasks (density and view-angle), each computed over the relevant portions of the test datasets.

#### 3.3.2. Auxiliary Task Contribution

To evaluate the contribution of each of the tasks (see section 2.2), we explored several task combinations: *breast*_bin_, *breast*_bin_ + rec, *breast*_bin_ + ACR, *breast*_bin_ + BIRADS, and *breast*_bin_ + view. We compared the prediction performance of the model with each combination vs. the full multi-task training considering all five tasks.

#### 3.3.3. Training on Few Data and Noisy Labels

To further evaluate the contribution of the studied tasks, we reduced the number of labels for *breast*_bin_. The goal of this experiment is to show that MTL improves the performance when fewer annotations are available or in presence of noisy labels. To this end we trained the MTL model, while randomly excluding 25, 50, and 75% of *breast*_bin_ labels (denoted as “*MTL*_breast−25%_,” “*MTL*_breast−50%_,” and “*MTL*_breast−75%_,” respectively) but keeping other available labels. We also trained the MTL model excluding 25, 50, and 75% of all labels (denoted as “*MTL*_all−25%_,” “*MTL*_all−50%_,” and “*MTL*_all−75%_,” respectively). For the state-the-art comparison, we train two mono-task methods using the baseline ResNet22 architecture.

The first method is the MixMatch pseudo-labeling technique (67, 68), which uses predictions of the pre-trained model as ground truth labels for further training (denoted as “MixMatch”). This method is designed to cope with the lack of labels. While the motivation is similar, their method follows a pseudollabeling instead of an MTL approach. We train the model on 50% of labels before generating the pseudo-label target values.

The second method is noisy labeling. Inspired by (69), we randomly exclude 50% of labels and then add noise to 50% and 100% of excluded labels (denoted as “*NL*_50%_” and “*NL*_100%_,” respectively). it allows simulating the noisiness of the labels coming from clinical practice (e.g., missing biopsy confirmation, false-negative diagnosis), hence evaluating the training under label noise scenario.

In these experiments, we trained the networks on *D*_HMI_train__ and tested on *D*_HMI_test__ and *D*_INB_

#### 3.3.4. Task-Specific Performances

We also explored the performance of the model individually trained for each task. Here we used the *E*(·) with multi-level feature fusion. We evaluated all four classification tasks: *breast*_bin_, ACR, BIRADS, and view. As before, we kept training on *D*_HMI_train__ and testing on *D*_HMI_test__ and *D*_INB_.

#### 3.3.5. Fine-Tuning With Uncertainty Scoring

Finally, we explored the proposed uncertainty-based loss weighting in the fine-tuning scenario. As a n initial model, we used the top-performing model pre-trained on *D*_HM_I__train__. For fine-tuning, we relied on the *D*_VTB_ dataset, which has heterogeneous labels, as described in section 2.3. In this dataset, most of the highly-graded ACR cases do not have a histopathology follow-up. Hence, there is an underlying uncertainty in the breast classification outcome, preventing the straightforward training of the binary classification task. To cope with the uncertainty, we, first, created synthetic *breast*_bin_ labels using the available ACR labels as follows: for the scores *ACR* ∈ {1, 2} we set *breast*_bin_ = 0, for *ACR* ∈ {4, 5} we set *breast*_bin_ = 1, *ACR* = 3 are ignored. Then, we defined the uncertainty scores *u*_*t*_ as described in section 2.1 for each sample. For simplicity of the experiments, we set the same uncertainty score *u*_1_*const*__ for all of the synthetic labels. We ran fine-tuning with several values of *u*_1_*const*__ ∈ {0.0, 0.25, 0.5, 0.75, 0.9} on *D*_VTB_ dataset and evaluated on *D*_HMI_test__ and *D*_INB_ (denoted *FT*_*u*_·__). For the labels of other tasks we set the uncertainty scores *u*_2.4_*const*__ = 0.5. We collected metrics for all the classifications tasks. In this experiment we also applied class reweighing in the loss function, to compensate for the significant under-representation of ACR4+ samples (see Table 1). We set the weight to 20 for all samples having *ACR* ∈ {4, 5}, and to 1 for all the other classes. We run 20 epochs of fine-tuning.

## 4. Results

With the experiments described in the previous section, we evaluate our proposed MTL method and report hereafter the results.

When **comparing to the state-of-the-art methods** (see Tables 2, 3) we achieve higher binary breast-cancer classification scores on both datasets, i.e., *AUC* = 80.46 and *AUC* = 78.13 on *D*_HMI_test__ and *D*_INB_, respectively, compared to the state-of-the-art methods. Noteworthy, our method yields more consistent classification performances in the stratified groups: there is less variation between binary classification scores when the evaluating performance on a subset of samples for for a given type of breast. Interestingly, our method outperforms (18) on both datasets (*AUC* = 80.46 vs. *AUC* = 71.26 and *AUC* = 78.13 vs. *AUC* = 74.04), while being trained on a smaller dataset. We note that we used the “image-only” method proposed by (18), which claims lower performances compared to other methods proposed by the authors. We hypothesize that our dataset might contain wider variety of mammography system vendors, favoring generalization.

**Table 2:** Evaluation on the test set.

	**Baseline**	**Wu et al. (18)**	**Ensembles**	**MC-drop**	**Ours (MTL)**
Overall	70.69 ± 0.36 (<0.01)	71.26 (<0.01)	73.39 ± 0.98 (<0.01)	72.82 ± 0.29 (<0.01)	80.46 ± 0.29
Density					
A	78.42 ± 0.12 (0.03)	81.34 (0.13)	79.14 ± 0.91 (0.05)	80.83 ± 0.10 (0.12)	87.26 ± 0.14
B	68.14 ± 0.19 (<0.01)	70.32 (<0.01)	72.14 ± 1.05 (<0.01)	70.97 ± 0.17 (<0.01)	79.58 ± 0.13
C	67.15 ± 0.16 (0.19)	68.46 (0.21)	68.23 ± 0.90 (0.23)	66.42 ± 0.16 (0.10)	74.23 ± 0.10
D	64.81 ± 0.09 (0.07)	74.81 (0.36)	67.12 ± 0.87 (0.11)	73.33 ± 0.19 (0.23)	83.44 ± 0.07
View angle					
CC	63.72 ± 0.14 (<0.01)	69.76 (<0.01)	67.54 ± 1.01 (<0.01)	66.27 ± 0.29 (<0.01)	78.47 ± 0.13
MLO	78.16 ± 0.21 (0.14)	69.82 (<0.01)	79.29 ± 1.12 (<0.30)	79.72 ± 0.29 (0.31)	82.44 ± 0.03

*Binary cancer classification performance [“AUC score (p-value to ours)”] of the compared methods on the entire D_HMI_test__ dataset and for the subsets filtered per density class and per view angle*.

**Table 3:** Generalization to an unseen dataset.

	**Baseline**	**Wu et al. (18)**	**Ensembles**	**MC-drop**	**Ours (MTL)**
Overall	72.45 ± 0.26 (0.02)	74.04 (<0.01)	73.34 ± 0.26 (0.02)	67.28 ± 0.26 (<0.01)	78.13 ± 0.63
Density					
A	74.30 ± 0.26 (0.28)	64.27 (<0.01)	72.15 ± 0.78 (0.03)	71.01 ± 0.22 (0.02)	78.91 ± 0.23
B	79.91 ± 0.35 (0.09)	80.23 (0.11)	77.43 ± 0.89 (0.07)	73.34 ± 0.13 (<0.01)	85.57 ± 0.32
C	55.05 ± 1.21 (0.11)	78.83 (<0.01)	56.64 ± 0.62 (0.13)	52.00 ± 0.11 (0.01)	65.61 ± 0.22
D	67.70 ± 1.09 (0.82)	62.50 (0.47)	64.39 ± 1.23 (0.62)	62.38 ± 0.17 (0.46)	70.83 ± 0.32
View angle					
CC	74.61 ± 0.73 (0.02)	72.43 (<0.01)	68.12 ± 1.46 (0.01)	63.79 ± 0.26 (<0.01)	83.06 ± 0.43
MLO	72.69 ± 0.47 (0.76)	79.83 (<0.01)	70.98 ± 1.83 (0.07)	70.20 ± 0.26 (0.05)	73.62 ± 0.37

*Binary cancer classification performance [“AUC score (p-value to ours)”] of the compared methods on the entire D_INB_ dataset and for the subsets filtered per density class and per view angle*.

We rely on the AUC metric to summarize the trade-off between the true-positive and the false-positive rates for different probability thresholds. The AUC is the most frequently reported score in the state-of-the-art (16–18, 32). Other metrics, such as sensitivity and specificity, require choosing an operating point. As an illustration, for the *D*_INB_ dataset, we obtain a sensitivity of 80.00% and a specificity of 49.03% when fixing the operating point at a malignancy probability of *p* > 0.5. In comparison, the method of (18) reaches a sensitivity of 2.00% and a specificity of 100.00% for the same operating point; while moving the operating point to *p* > 0.02 leads to scores of 75.00 and 53.87, respectively. We observe a similar behavior on the *D*_HM_I__test__ dataset. Using the same operating point (*p* > 0.5), our method obtains scores of 79.92 and 68.63, comparable to those for *D*_INB_. However, for the method from (18), the operating point of *p* > 0.02 leads to scores of 65.84 and 62.45. We also explored the Matthews correlation coefficient (MCC) for its better fitness to the unbalanced-dataset scenario, such as *D*_INB_ (70). In our case, we obtained the highest *MCC* = 0.53, at Sensitivity of 51.00 and Specificity of 94.52. The baseline method yields *MCC* = 0.43 at Sensitivity of 53.00 and Specificity of 88.06. The classifier of (18) gives highest *MCC* = 0.41 at Sensitivity of 44.00 and Specificity of 91.61.

We were also interested in the triage scenario, where the classifier could safely predict the benign and normal cases with a low false negatives rate. Hence, we compared the operating point at 95% of sensitivity. For the proposed method, we obtained *MCC* = 0.24 and a Specificity of 29.03. For the baseline we obtained *MCC* = 0.03 and Specificity of 6.45, and for (18) we obtained *MCC* = 0.18 and Specificity of 21.55. While the performances are yet to achieve reliable medical diagnosis performances, we observe a positive trend with our proposed method, showing the potential of safely classifying almost 30% of samples as benign.

When evaluating the **contribution of the auxiliary tasks** (see Table 4), the largest improvement is brought by the reconstruction (“rec”) task (*AUC* = 78.09 ± 1.06 with a 7.4% gain), followed by the *ACR* classification tasks (*AUC* = 77.60 ± 0.26 with a 6.91% gain). Nevertheless, the *view* and *BIRADS* classes also contribut e, allowing to achieve scores of *AUC* = 74.45 ± 0.81 and *AUC* = 76.38 ± 0.92, respectively.

**Table 4:** Evaluation of each auxiliary task contribution to the binary cancer classification on the *D*_HMI_test__ and *D*_INB_ datasets; “+” stands for enabled task and “−” stands for disabled task.

**Task**					**Dataset**	
**breast_bin_**	**rec**	**ACR**	**BIRADS**	**view**	***D*_HMI_test__**	***D*_INB_**
+	+	+	+	+	80.46 ± 0.79	78.13 ± 0.83
+	−	−	−	−	70.69 ± 0.36	72.45 ± 0.26
+	+	−	−	−	78.09 ± 1.06	76.47 ± 0.31
+	−	+	−	−	77.60 ± 0.26	77.03 ± 0.78
+	−	−	+	−	74.45 ± 0.81	73.97 ± 0.54
+	−	−	−	+	76.38 ± 0.92	76.67 ± 1.07

*The AUC score is reported*.

We evaluated the results of the MTL when **reducing the number of samples** vs. excluding only the breast_bin_ labels. We note that keeping the auxiliary labels allows maintaining higher performances (see Table 5). Remarkably, the performance of the baseline model (see Tables 2, 3), trained on the full *D*_HM_I__train__ dataset with complete labels is lower than our MTL with only 50% of samples retained. In the same set of experiments, we also compare to two state-of-the-art methods (see section 3.3.3) dealing with few annotated samples, noisy labels, or under uncertain labels (see Table 5). Our method achieved higher *AUC* scores, although statistical significance was not systematically verified.

**Table 5:** Evaluation of the capabilities of auxiliary tasks to improve the binary breast classification performed on the *D*_HMI_test__ and *D*_INB_ datasets, while training with fewer cases annotated with *birads*_bin_ labels.

	**Dataset**	
	***D*_HMI_test__**	***D*_INB_**
Reference	80.46 ± 0.79	78.13 ± 0.83
*MTL*_breast−25%_	78.53 ± 0.22	76.01 ± 1.02
*MTL*_breast−50%_	78.57 ± 0.19	74.07 ± 1.11
*MTL*_breast−75%_	76.20 ± 0.39	71.89 ± 0.87
*MTL*_all−25%_	77.85 ± 0.50	77.01 ± 0.98
*MTL*_all−50%_	76.37 ± 0.49	72.32 ± 0.78
*MTL*_all−75%_	74.18 ± 0.37	69.97 ± 0.92
MixMatch	74.85 ± 0.44	75.10 ± 0.86
*NL*_50%_	73.82 ± 0.56	72.88 ± 0.77
*NL*_100%_	72.12 ± 0.67	74.55 ± 1.21

*AUC score is reported*.

We separately evaluated the **role of the multi-level feature fusion** component. When training on the unique binary classification task with feature fusion we obtain an *AUC* = 75.07 ± 0.56 on *D*_HMI_test__, which is superior than the *AUC* = 70.69 ± 0.36 obtained without the fusion. The best performance is however attained when combining the feature fusion with the MTL training (i.e., *AUC* = 80.46 ± 0.29).

We compared the performance of our MTL approach to the mono-task-trained networks, considering each of the 4 classification tasks (see Tables 6, 7). We observe that MTL improves the performance for binary breast cancer and *ACR* classification tasks. For the unseen distribution (i.e., *D*_INB_), the scores sometimes decrease as in case of density and view-angle classification. That is, we obtain an accuracy of 31.70 vs. 48.75 for the density classification and *AUC* = 84.12 vs. *AUC* = 97.28 for the view-angle classification. In this work, we report weighted accuracy for ACR and density multi-class classification. We additionally explored other metrics, such as the F1-score for these tasks observing similar trends. For the *D*_HMI_test__ dataset, we obtain *F*_1_ = 65.95 ± 1.05 for MTL and *F*_1_ = 64.35 ± 0.46 for the mono-task training in density classification task. Similarly, for the *D*_INB_ dataset, we obtain *F*_1_ = 32.11 ± 1.87 and *F*_1_ = 48.92 ± 1.07 for the MTL and mono-tasks trainings, respectively.

**Table 6:** Comparison of the multi-task training to the mono-task on the *D*_HMI_test__ dataset; “-” stands for unavailable metrics.

	**Task**			
	**breast_bin_**	**ACR**	**BIRADS**	**View**
	***AUC***	***Accuracy***	***Accuracy***	***AUC***
*MTL*	80.46 ± 0.79	55.95 ± 1.12	67.66 ± 1.06	96.55 ± 0.70
*breast*_bin_	70.69 ± 0.36	-	-	-
ACR	-	54.67 ± 1.13	-	-
BIRADS	-	-	65.92 ± 0.96	-
view	-	-	-	97.14 ± 0.46

**Table 7:** Comparison of the multi-task to the mono-task on the *D*_INB_ dataset; “-” stands for unavailable metrics.

	**Task**			
	**breast_bin_**	**ACR**	**BIRADS**	**View**
	***AUC***	***Accuracy***	***Accuracy***	***AUC***
*MTL*	78.13 ± 0.83	58.29 ± 1.41	31.70 ± 2.32	84.12 ± 1.32
*breast*_bin_	72.45 ± 0.26	-	-	-
ACR	-	15.60 ± 2.10	-	-
BIRADS	-	-	48.75 ± 1.23	-
view	-	-	-	97.28 ± 0.98

Our fine-tuning experiments show the potential of our proposed approach to learn from datasets with scarce or noisy labels. The performance increase is particularly relevant for the experiment with the unseen dataset (*D*_INB_), where an improvement w.r.t the initial model is visible for all metrics (see Tables 8, 9).

**Table 8:** Effect of uncertainty-based training with *breast*_bin_ labels generated from ACR-classification labels on *D*_VTB_ dataset.

	**Task**			
	**breast_bin_**	**ACR**	**BIRADS**	**View**
	***AUC***	***Accuracy***	***Accuracy***	***AUC***
Reference (MTL)	80.46 ± 0.79	55.95 ± 1.12	67.66 ± 1.06	96.55 ± 0.70
*FT*_*u*_0.00__	72.67 ± 0.85	51.78 ± 1.43	63.11 ± 1.01	95.01 ± 1.20
*FT*_*u*_0.25__	73.15 ± 0.64	52.13 ± 1.52	62.29 ± 1.11	96.01 ± 0.99
*FT*_*u*_0.50__	74.23 ± 0.91	53.97 ± 0.88	65.09 ± 1.02	95.96 ± 0.98
*FT*_*u*_0.75__	79.32 ± 0.73	53.23 ± 1.18	65.97 ± 0.87	96.07 ± 1.09
*FT*_*u*_0.90__	80.62 ± 0.59	56.01 ± 1.09	66.49 ± 0.98	96.13 ± 1.10

*The evaluation is performed on the D_HMI_test__ dataset*.

**Table 9:** Effect of uncertainty-based training with breast_bin_ labels generated from ACR-classification labels on D_VTB_ dataset.

	**Task**			
	**breast_bin_**	**ACR**	**BIRADS**	**View**
	**AUC**	**Accuracy**	**Accuracy**	**AUC**
Reference (MTL)	78.13 ± 0.83	58.29 ± 1.41	31.70 ± 2.32	84.12 ± 1.32
FT_u_0.00__	72.24 ± 0.83	55.12 ± 1.92	31.54 ± 1.11	85.32 ± 1.05
FT_u_0.25__	74.42 ± 0.92	57.03 ± 1.21	30.10 ± 2.04	89.33 ± 1.44
FT_u_0.50__	76.85 ± 0.71	60.08 ± 2.04	36.54 ± 1.67	92.01 ± 1.23
FT_u_0.75__	78.42 ± 0.90	61.97 ± 1.42	37.94 ± 1.03	92.16 ± 0.87
FT_u_0.90__	81.40 ± 0.45	62.68 ± 1.01	38.54 ± 1.24	91.98 ± 0.97

*The evaluation is performed on the D_INB_ dataset*.

## 5. Discussion

In this work, we proposed an MTL strategy to cope with the missing and uncertain labels of mammograms while addressing the binary breast classification. We departed from the difficulty of patient tracking preventing the collection of confident labels for a fully-supervised learning (e.g., missing follow-ups, lacking biopsy information). To address this issue, we proposed several auxiliary tasks that increase the amount of data eligible for training deep-learning-based algorithm s at a low er cost of data mining and annotation. Our approach enables the use of labels available from clinical reports and patient cases, such as the BI-RADS breast density classes, the ACR cancer probability, and the view angle. We also proposed to deal with labels uncertainty through loss weighting at training time.

The design of our deep neural network architecture contains three main components: (i) a feature fusion block combining features from multiple levels of a ResNet-like encoder; (ii) a light-weight decoder for image reconstruction playing the role of implicit regularizer; and (iii) 4 classification outputs from the bottleneck of the auto-encoder. All three components contribute concurrently to the improvement of the performances. Moreover, our uncertainty-weighted training strategy has also shown the potential to improve the classification performance while training on heterogeneous data. In this case, the improvement is conditioned to a high uncertainty label (i.e., 0.90). We attribute this phenomenon in part to the significant data imbalance (see Table 1) and in part to the eventual presence of noisy labels (false positives and false negatives) in the dataset.

Our results (see section 4) demonstrate the statistically significant superiority of our method compared to the baseline and state-of-the-art methods, such as MC-Dropout, Ensembles, as well a top-performing classification method (18).

Besides the performance improvement in several cases, our method allows for a processing time gain compared to the mono-task method s. That is, our network has the advantage of performing several clinically relevant tasks (for example, binary cancer and density classifications) in one forward pass, while in the case of mono-task networks, one pass per task is needed. The proposed architecture could be further extended to a MC-Dropout- or ensemble-type setup. Since it will lead to a longer processing or/and larger models, we did not explore this scenario in the present study. However, it can be part of future work.

We note that our MTL method does not reach the state-of-the-art performances claimed by (16) and (17), i.e., *AUC* = 95.0 on the INBreast dataset. However, both of these methods rely on additional labels provided by experts in the form of ROI around malignant/suspicious regions. These ROI are used for region detection in case of (16), and for patches extraction in case of (17). Moreover, the metrics claimed by (17) are collected only on a portion of the dataset, unlike our metrics computed on the entire dataset. Furthermore, as our method is designed to produce an image-wise prediction, we compute image-wise metrics. If we instead follow the evaluation protocol in (16), computing breast-wise predictions (i.e., considering the highest malignancy probability for two views of a breast), we obtain an *AUC* = 85.23.

**Figure 6:**
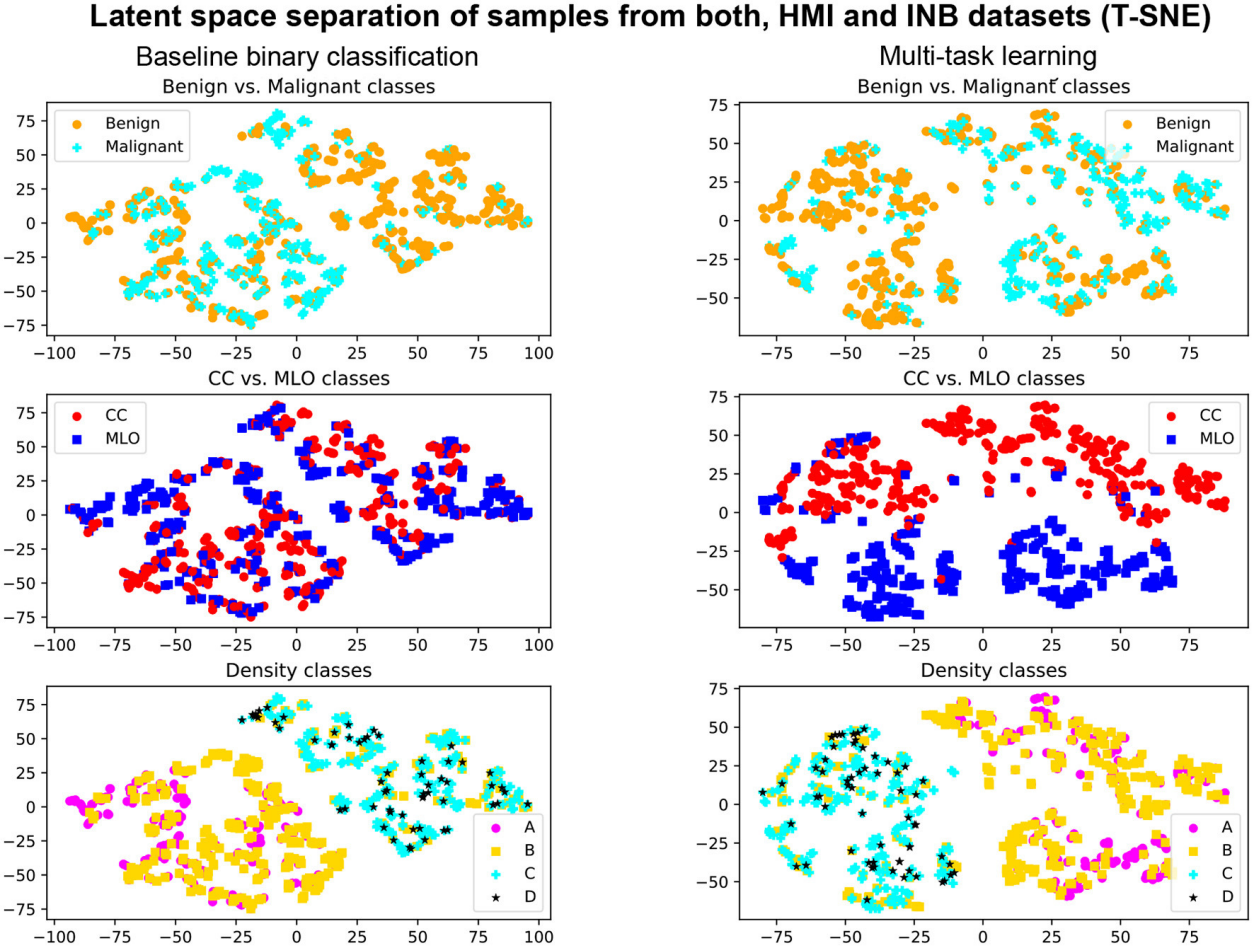
Illustration T-SNE representation of the samples from *D*_HM_I__test__ and *D*_INB_ datasets with Multi-Task Learning **(right)** and without **(left)**. First row: separation of benign and malignant classes. Second row: separation of CC and MLO views. Third row: separation of density classes.

The MTL approach also contributes to the explainability of the method. Indeed, the simultaneous training allows for better-shaped latent representation at the auto-encoder bottleneck (see Figure 6). We, therefore, expect that samples fall closer to anatomically- and physiologically-related images, even if misclassified or uncertain. We also observe better performances with the multi-task model when evaluating specific types of breast densities or view angles, while the network is trained on the entire dataset (see Tables 2, 3).

Our approach is relatively straightforward, allowing for the introduction of other tasks, e.g., classification upon the presence of different findings, such as masses, calcifications, etc. This offers a potential of performance improvement at little cost.

Thanks to the MTL approach, our method can ease learning in environments where all the labels are not easily available, for example (if the regulation permits) in a federated learning setup (71, 72). In this context, the network could be trained from a continuous flow of imaging even eventually without dealing with clinical reports: e.g., labels, such as view angle, can be retrieved from imaging meta-data, while density annotations can be generated with pseudo-labeling.

## 6. Conclusion

This work is a contribution to the field of breast cancer classification research. The proposed method aims to provide a reliable prediction of breast cancer using mammography images as input. To improve the quality of the prediction, we propose to rely on multi-task learning, introducing several tasks, including in particular density and view angle classification. We observe the superiority of our method compared to other state-of-the-art approaches when evaluating on two independent datasets. Moreover, we note a more meaningful representation of the images in the latent space supporting the explainability of the method.

Despite the performance gain, our method still offers room for improvement. First, there is still a gap w.r.t. to state-of-the-art methods using finer levels of annotations [*AUC* = 95.00 in (17) and (16)], i.e., relying on labels at the pixel or region level instead of only image-wise in our case. Second, the marginal improvement of the uncertainty-based loss-weighting is probably due to our naive determination of the uncertainty weights; measuring or better modeling the uncertainties could lead to a larger performance impact. Moreover, our metrics show that there is still a gap to fill toward a clinically acceptable medical diagnostic solution. That is, we are facing a choice of high specificity or high sensitivity, having to sacrifice the sensitivity or specificity, respectively. We note, however, that in the high-sensitivity setup, our method allows for cases triage with a low false-negative rate: we achieve 30% specificity at 95% sensitivity on *D*_INB_, and similarly, 37% specificity with 95% sensitivity on *D*_HM_I__test__. The improvements of classification performances may be achieved, for example, in ensembles models setup, which could be part of future explorations.

Future work could also include a stronger uncertainty modeling, in particular relying on the prediction uncertainty as in (55), instead of the prior knowledge on the dataset. Another possible direction is the extension of the auxiliary tasks performed by the network by introducing, for example, a segmentation task in a self-supervised scenario as in (28). Moreover, in this work simplified the loss balancing by multiplying each task loss with the factor of 1. Other balancing weights, based on experiments or learned dynamically, could be studied in the future. Finally, the explainability could be improved with a more explicit shaping of the latent space enforced through modeling constraints.

Overall, our work presents a step forward in the direction of more reliable cancer classification and opens several paths for future research.

## Data Availability Statement

Two publicly available datasets were used in this work. Data from the Susan G. Komen Tissue Bank at the IU Simon Cancer Center were used in this work. This data is available online http://virtualtissuebank.iu.edu/. We used a subset of the dataset. The list of used cases can be obtained by the sending a request to the corresponding author. Data from the INBreast dataset (30) were also used. The data can be accessed by the sending a request to the authors. Furthermore, a multi-site private dataset was used in this work. Private agreements were signed between the institutions and Hera-MI. Institutional board approvals were obtained from each of the sites. This dataset can be shared upon justified requests and subsequent rights-holder approvals. Requests are to be addressed to the corresponding author. The trained models used in the experiments can also be shared in the absence of conflict of interest and stakeholders approvals. Requests are to be addressed to the corresponding author.

## Author Contributions

MT is the first author of this paper. DM is the senior author of this paper. All authors contributed to the article and approved the submitted version.

## Funding

This work was supported by Hera-MI SAS, ANRT grant 2018/0308, and the European Regional Development Fund, Pays de la Loire and Nantes Métropole (Connect Talent MILCOM).

## Conflict of Interest

MT is employed by Hera-MI as Chief Scientific Officer. The remaining author declares that the research was conducted in the absence of any commercial or financial relationships that could be construed as a potential conflict of interest.

## Publisher's Note

All claims expressed in this article are solely those of the authors and do not necessarily represent those of their affiliated organizations, or those of the publisher, the editors and the reviewers. Any product that may be evaluated in this article, or claim that may be made by its manufacturer, is not guaranteed or endorsed by the publisher.

## Footnotes

^1^https://gco.iarc.fr/today/.

^2^https://github.com/nyukat/breast_cancer_classifier/.
